# *Caenorhabditis elegans *Muscleblind homolog *mbl-1 *functions in neurons to regulate synapse formation

**DOI:** 10.1186/1749-8104-7-7

**Published:** 2012-02-07

**Authors:** Kerri A Spilker, George J Wang, Madina S Tugizova, Kang Shen

**Affiliations:** 1 Department of Biology, Howard Hughes Medical Institute, Stanford University, 385 Serra Mall, Stanford, California 94305, USA

## Abstract

**Background:**

The sequestration of Muscleblind splicing regulators results in myotonic dystrophy. Previous work on Muscleblind has largely focused on its roles in muscle development and maintenance due to the skeletal and cardiac muscle degeneration phenotype observed in individuals with the disorder. However, a number of reported nervous system defects suggest that Muscleblind proteins function in other tissues as well.

**Results:**

We have identified a mutation in the *Caenorhabditis elegans *homolog of Muscleblind, *mbl-1*, that is required for proper formation of neuromuscular junction (NMJ) synapses. *mbl-1 *mutants exhibit selective loss of the most distal NMJ synapses in a *C. elegans *motorneuron, DA9, visualized using the vesicle-associated protein RAB-3, as well as the active zone proteins SYD-2/liprin-α and UNC-10/Rim. The proximal NMJs appear to have normal pre- and postsynaptic specializations. Surprisingly, expressing a *mbl-1 *transgene in the presynaptic neuron is sufficient to rescue the synaptic defect, while muscle expression has no effect. Consistent with this result, *mbl-1 *is also expressed in neurons.

**Conclusions:**

Based on these results, we conclude that in addition to its functions in muscle, the Muscleblind splice regulators also function in neurons to regulate synapse formation.

## Background

Myotonic dystrophy type 1 (DM1) is a disorder characterized by progressive muscle degeneration and myotonia, or the failure of muscles to relax. The underlying molecular cause of DM1 is an expansion of CTG-repeats in the 3' untranslated region of the gene *DMPK *(dystrophia myotonica-protein kinase) [1]. These expanded CUG-repeats bind with high affinity to the Muscleblind-like (MBNL) alternative splicing regulators and result in the sequestration of MBNL proteins in the nucleus [2,3], rendering them unable to perform their normal splicing functions and disease phenotypes are due partly to changes in splicing of MBNL target genes (reviewed in [4]).

All metazoans have at least one MBNL-type splicing regulator, each with two or four zinc-finger CCCH RNA-binding domains in the amino-terminal half of the protein [5]. In crystal structures, the zinc fingers bind to specific RNA sequences and it is hypothesized that MBNL proteins regulate splicing by binding directly to RNA [6].

Because MBNL proteins are enriched in muscle tissue and skeletal and cardiac muscle defects are prominent features of DM1, most studies of MBNL genes have focused on their roles in muscle development and maintenance. Previous work demonstrated that MBNL proteins are responsible for the proper splicing of the muscle-specific chloride channel (Clcn1) and mis-splicing of this gene is responsible for the muscle myotonia observed in individuals with DM1 [7].

Muscle defects are also observed in other organisms lacking MBNL genes. In the absence of Mbnl1 or Mbnl2, mice exhibit both muscle abnormalities and myotonia [8,9]. Loss of *Drosophila *Muscleblind is lethal at the larval stage due to ultrastructural muscle defects [10] and RNA interference against the *Caenorhabditis elegans *MBNL homolog *mbl-1 *resulted in muscle disorganization defects [11]. Microarray studies have revealed that over 100 genes in mouse muscle tissue are mis-spliced in the absence of mouse Mbnl1 [12].

Historically, MBNL studies have focused on the role of MBNL in muscle; however, several studies have hinted that MBNL may also function in the nervous system. MBNL expression has been observed in human and mouse brain [5,13], chick photoreceptor neurons [14], zebrafish brain and spinal cord [15], and the *Drosophila *embryonic ventral nerve cord [10]. MBNL1 expression has been observed in the cytoplasm and nuclei in human spinal cord motorneurons [16]. Loss of *Drosophila *Muscleblind causes photoreceptor differentiation defects [17] and humans with DM1 suffer from a range of cognitive and behavioral deficits, including mental retardation and behavioral disorders [18].

We identified a *C. elegans *mutant with a defect in synapse formation and mapped the mutation to a locus containing the MBNL homolog *mbl-1*. The nervous system of *C. elegans *is composed of 302 neurons that form approximately 5,000 synapses and 2,000 gap junctions in a highly stereotyped and reproducible pattern [19]. Using cell-specific promoters and fluorescently tagged synaptic vesicle-associated proteins in this well-characterized system, we are able to observe synapses form in individual neurons. In *mbl-1 *mutants, a subset of synapses in the dorsal nerve cord fail to form. Interestingly, we found that the neuromuscular junction (NMJ) defect of *mbl-1 *can be rescued by neuronal expression, but not by muscle expression of *mbl-1*, suggesting that MBL-1 functions in neurons to regulate synapse formation.

## Results

### Loss of *mbl-1 *alters synapse formation in the cholinergic motorneuron DA9

Located in the preanal ganglion, the cholinergic motorneuron DA9 is the most posterior of the DA-type motorneurons with a dendrite that extends anteriorly from the cell body and an axon that extends posteriorly, then crosses the midline of the animal via a commissure and extends anteriorly in the dorsal nerve cord (DNC). While in the DNC, DA9 forms approximately 25 *en passant *dyadic synapses onto body wall muscles and reciprocal inhibitory VD neurons [20].

**Figure 1:**
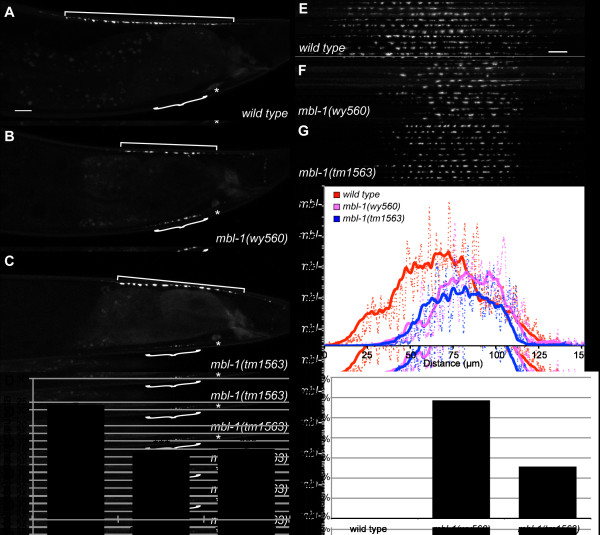
***mbl-1 *disrupts synaptic vesicle localization in the motorneuron DA9. (A-C) **Localization of synaptic vesicle-associated GFP::RAB-3 in adult wild type (*wyIs85*) (A), *mbl-1(wy560) *(B) and *mbl-1(tm1563) *(C) mutants. Animals are oriented such that anterior is to the left and dorsal is up. Synaptic region (square bracket), dendrite (curly bracket) and DA9 cell body (asterisk) are indicated. Scale bar represents 10 μm. **(D) **Quantification of the number of axonal RAB-3 puncta observed by epifluorescence microscopy. Error bars represent standard error of the mean. ***P < 0.0001, *t*-test. **(E-G) **Confocal micrographs of ten wild-type (*wyIs85*), *mbl-1(wy560) *or *mbl-1(tm1563) *animals, straightened and aligned along their anteroposterior axes. Scale bar equals 10 μm. Anterior is to the left. **(H) **Quantification of fluorescence intensity along the x-axis of the composite images. Dotted lines represent actual values and solid lines represent a 10-μm sliding average of fluorescence intensity. **(I) **Percentage of animals with visible puncta in the dendrite in wild-type, *mbl-1(wy560) *and *mbl-1(tm1563) *animals.

To study synapse formation in DA9, we used a cell-specific promoter to express a green fluorescent protein (GFP)-tagged version of the synaptic vesicle associated protein RAB-3 (GFP::RAB-3) [21]. In wild-type animals, RAB-3 accumulates in discrete puncta along the axon of DA9 at stereotyped locations and co-localizes with presynaptic active zone proteins. These puncta correspond to the location of synapses in electron microscopy reconstructions of DA9 [19,22] (Figure 1A).

**Figure 2:**
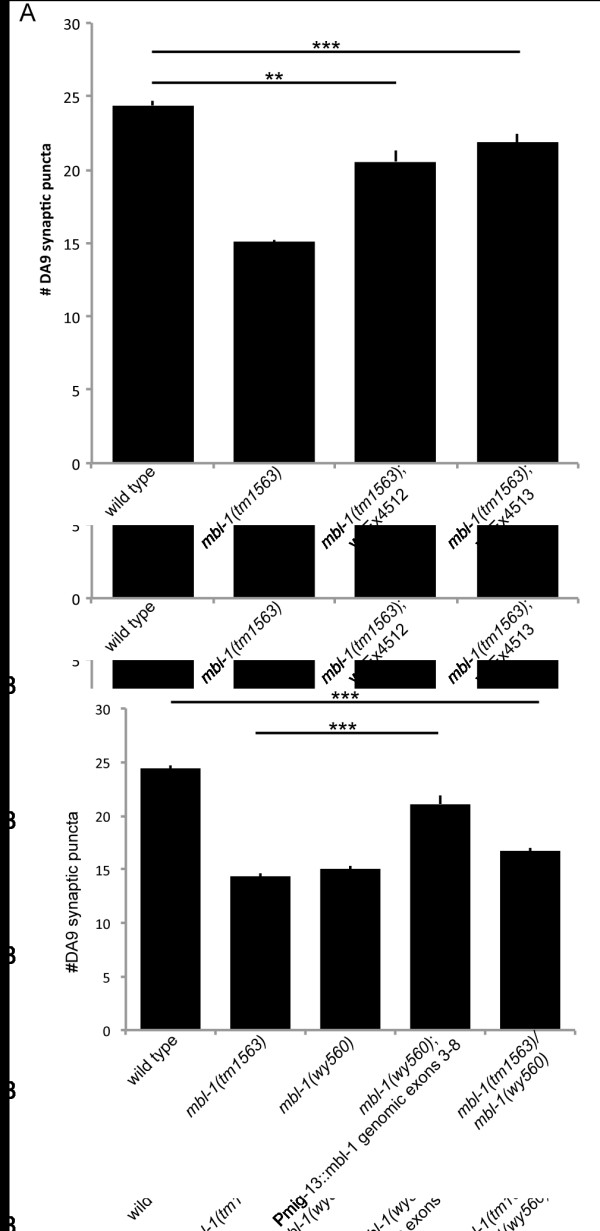
**Quantification of rescue of DA9 synaptic defect. (A) **Quantification of the rescue of the DA9 synapse defect in *mbl-1(tm1563) *mutants with fosmid WRM0616bE04 tagged with GFP::SL2::mCherry (*wyEx4512 *and *wyEx4513*). Error is standard error of the mean. **P < 0.001, ***P < 0.0001. **(B) **Rescue of DA9 synapse number in *mbl-1(wy560) *with tagged fosmid (KAS138-5). Trans-heterozygous *mbl-1(tm1563)*/*mbl-1(wy560) *animals have a similar phenotype to homozygous mutants.

While working with the strain RB771, we identified an unmapped locus that disrupts the normal pattern of synapses in DA9. Using SNP polymorphisms between N2 and Hawaiian strains [23], we mapped this mutation to the far right arm of the X chromosome. After whole genome sequencing we identified a 70-kb deletion that eliminates eight genes, one of which is *mbl-1*. Since the *wy560 *mutation completely eliminates the *mbl-1 *locus, it represents a null allele for this gene. A second allele, *mbl-1(tm1563)*, is a 513-bp deletion that eliminates all of exon 3 of *mbl-1*, an exon shared among all *mbl-1 *isoforms. Exon 3 skipping is predicted to cause an in-frame deletion of some isoforms; however, for the three isoforms that utilize a start codon in exon 3, the next downstream start codon would result in a frame-shift. Further analysis revealed that trans-heterozygous *mbl-1(tm1563)*/*mbl-1(wy560) *animals have a synapse number phenotype identical to that of the homozygous single mutants (Figure 2) and because the two alleles of *mbl-1 *showed an indistinguishable defect for DA9 synapse number, the *tm1563 *allele is likely acting as a null with respect to the DA9 synapse number phenotype.

In both *mbl-1(wy560) *and *mbl-1(tm1563) *mutant animals, several aspects of DA9 presynaptic patterning are disrupted (Figure 1). The most penetrant phenotype is the loss of approximately ten synapses from the DA9 synaptic region: 24.4 ± 3.6 RAB-3 puncta in wild-type animals versus 14.3 ± 2.2 in *mbl-1(wy560) *mutants and 15.0 ± 2.4 in *mbl-1(tm1563) *mutants (standard deviation, *P *< 0.0001, *t*-test; Figure 1D). To quantitatively analyze this phenotype, we imaged the DA9 synaptic region in ten wild-type and mutant animals by confocal microscopy and constructed composite images of the presynaptic regions of all ten animals by aligning them along their anteroposterior axes (Figure 1E-G). We observe that the most distal GFP::RAB-3 puncta are absent in *mbl-1(wy560) *and *mbl-1(tm1563)*. However, the spacing between the remaining synaptic puncta in both *mbl-1(wy560) *and *mbl-1(tm1563) *is similar to the spacing between GFP::RAB-3 puncta in wild-type animals. Examination of a transgenic line (*wyEx1902*) in which DA9 is labeled with a cytoplasmic fluorophore demonstrates that DA9 neuronal morphology is normal in *mbl-1(tm1563) *and loss of these distal synapses is not a result of axonal truncation (data not shown.)

Measurement of the total fluorescence intensity of the composite images along the x-axis demonstrates that the peak fluorescence is indeed more posterior in *mbl-1(wy560) *and *mbl-1(tm1563) *mutant animals compared to wild type (Figure 1H). Furthermore, it appears that the highest point of fluorescence intensity is lower in *mbl-1(wy560) *and *mbl-1(tm1563)*. Thus, in *mbl-1 *mutants, fewer synapses form and the synapses that are present seem to contain less RAB-3 than their wild-type counterparts.

Imaging analysis of *mbl-1(wy560) *and *mbl-1(tm1563) *mutants also revealed the presence of ectopic RAB-3 in compartments of DA9 from which RAB-3 is normally excluded. In 59% of *mbl-1(wy560) *and 26% of *mbl-1(tm1563) *animals, we observe puncta in the DA9 dendrite, compared to 0% for wild type (Figure 1I). When visible, these ectopic dendritic puncta are significantly smaller than dorsal synaptic puncta and mutants typically have one to six puncta. We also observe ectopic puncta in other segments of DA9, including the posterior dorsal asynaptic region, commissure, and ventral axon.

### Other pre-synaptic markers are affected in *mbl-1*, but post-synaptic markers are unchanged

**Figure 3:**
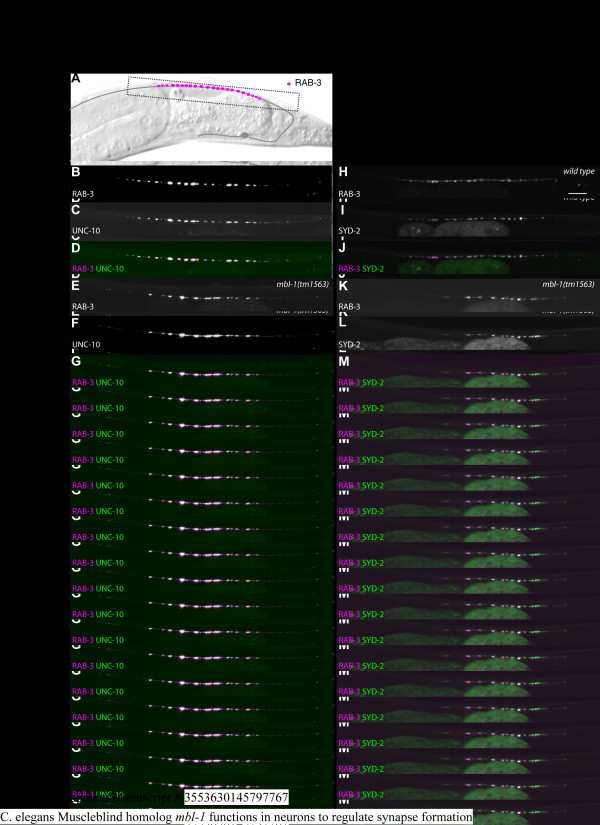
**Localization of pre-synaptic proteins is disrupted in *mbl-1 *mutants. (A) **Schematic of DA9 and wild-type localization of RAB-3. **(B-G) **Co-localization of RAB-3 and UNC-10 in wild type (B-D) and *mbl-1(tm1563) *(E-G). *(wyEx3709)*. **(H-M) **RAB-3 and the active zone protein SYD-2 in wild type (H-J) and *mbl-1(tm1563) *(K-M). Transgene used is *wyEx2055*. Scale bar represents 10 μm.

The RAB-3 phenotype in *mbl-1 *mutants prompted us to ask whether this defect is a result of impaired synaptic vesicle transport or a failure of *de novo *synapse formation by analyzing the localization of other pre-synaptic proteins. The presynapse is a complex structure composed of many active zone proteins required to facilitate the exocytosis of synaptic vesicles in response to an action potential, as well as vesicle-associated proteins that allow active zone machinery to interact with synaptic vesicles [24]. We expressed two fluorescently tagged active zone proteins, UNC-10 (Rim) [25] and SYD-2 (Liprin-α) [26], in DA9 and examined their localization in the wild-type and *mbl-1(tm1563) *DA9 synaptic regions (Figure 3A). We find that UNC-10::GFP co-localizes with mCherry::RAB-3 at DA9 synapses in both wild-type and *mbl-1(tm1563) *animals, though there are fewer puncta overall in *mbl-1(tm1563) *mutant animals compared to wild-type animals (Figure 3B-G). Similarly, we find that GFP::SYD-2 and mCherry::RAB-3 co-localize, but there are again fewer total DA9 presynaptic puncta in *mbl-1(tm1563) *(Figure 3H-M). Taken together, these data suggest the defect in *mbl-1(tm1563) *represents the failure of synapse formation in the most distal part of the DA9 synaptic region.

**Figure 4:**
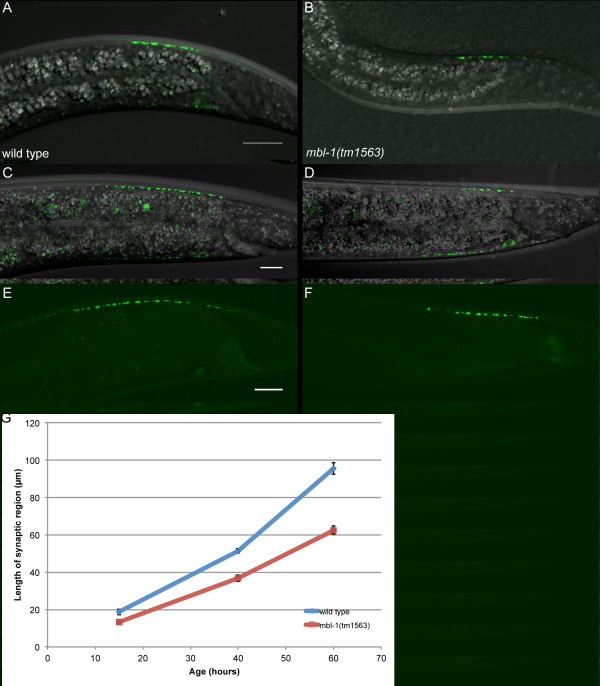
**Measurement of synapse length. (A-D) **Representative images of DA9 synapses labeled with SNB-1::YFP in animals that are approximately 15 hours old and at the L2 stage (A, B) and approximately 40 hours old and at the L4 stage (C, D). Transgene used is *wyIs92*. **(E-F) **Images of GFP::RAB-3 in early-stage adult animals. Trasgene used is *wyIs85*. **(G) **Quantification of synapse length at three stages for wild-type (blue) and *mbl-1(tm1563) *(red) animals.

To clarify whether the DA9 defect we observe is the result of improper synapse maintenance during development or an acute loss of synapses at the adult stage, we measured the length of the DA9 synaptic region in animals at the L2, L4, and adult stages (Figure 4). We measured the synaptic length rather than counting individual puncta because puncta are densely clustered in young animals, making it difficult to count individual punctum. We observe that there is a consistently shorter synaptic region in DA9 in *mbl-1(tm1563) *at all timepoints measured. This absolute difference translates to a 28% decrease in synapse length at the L2 stage, a 28% decrease at the L4 stage, and a 35% decrease in young adults. These results demonstrate that *mbl-1 *mutants animals consistently fail to accumulate the appropriate amount of synaptic material at the DA9 synaptic region throughout development.

We next asked whether the change in DA9 synaptic patterning is accompanied by changes in the postsynaptic muscle partner as loss of MBNL in human, mouse, and *Drosophila *all result in muscle changes. DA9 forms dyadic synapses onto body wall muscles and VD inhibitory motorneurons. Upon DA9 acetylcholine release, acetylocholine binds to postsynaptic acetylcholine receptors on body wall muscles and causes contraction of dorsal body wall muscles. The *C. elegans *acetylcholine receptor, ACR-16, is important for excitatory neuronal transmission at the NMJ [27] and localizes in a discrete line along the dorsal body wall muscles.

**Figure 5:**
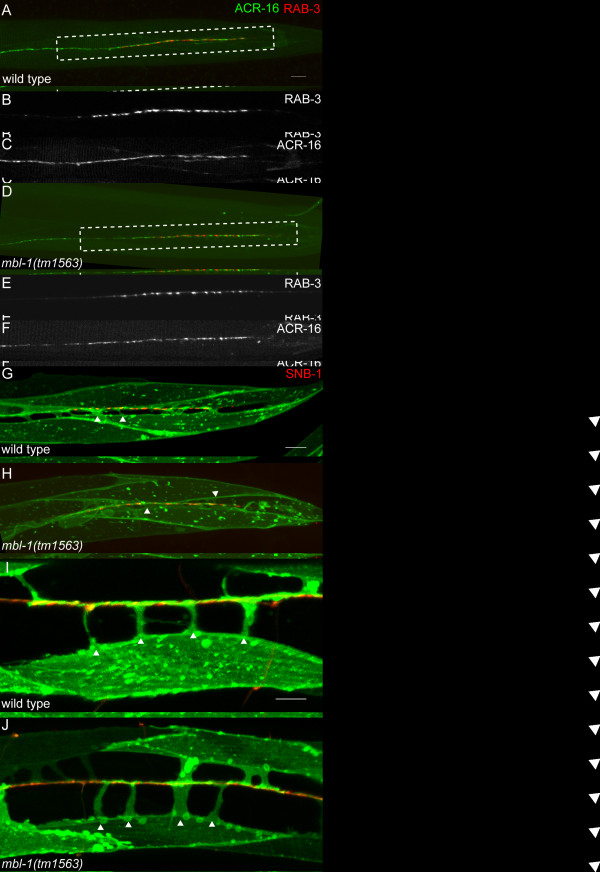
**Localization of post-synaptic receptor ACR-16 and body wall muscle morphology is normal in *mbl-1*. (A-F) **Postsynaptic receptor ACR-16::GFP co-localizes with presynaptic mCherry::RAB-3 in wild type (A-C) and *mbl-1(tm1563) *(D-F) mutants in the dorsal nerve cord. ACR-16 is shown in green and RAB-3 is shown in red. **(G, H) **Dorsal body wall muscles labeled with myr-mCherry extend muscle arms towards the dorsal nerve cord where DA9 synapses are labeled with SNB-1::YFP. Muscles and SNB-1 are pseudocolored green and red, respectively. Two representative muscle arms are indicated with a white triangle for each genotype. **(I, J) **Lateral body wall muscles labeled with membrane-tethered yellow fluorescent protein (YFP; green) extend muscle arms towards the dorsal nerve cord labeled with mCherry (red). Four muscle arms, each indicated by a white triangle, are observed per muscle for both wild type and *mbl-1(tm1563)*.

We examined ACR-16::GFP localization in wild-type and *mbl-1(tm1563) *mutants (Figure 5A-F). ACR-16:GFP localization was qualitatively similar between wild-type and mutant animals, with relatively continuous staining along the DNC. ACR-16 staining appears to cover more area compared with the RAB-3. This is to be expected because the ACR-16 staining represents postsynaptic specializations with both DA and DB classes of neurons, while the RAB-3 staining is only in the DA9 neuron. Qualitative observation of mCherry::RAB-3 puncta in both wild-type and *mbl-1(tm1563) *worms reveals that most RAB-3 co-localizes with ACR-16::GFP in the DNC (data not shown).

Previous reports have documented muscle defects in *mbl-1 *mutant animals [11]. We examined muscle morphology in two transgenic lines in which muscles are labeled with a cytoplasmic fluorophore, *wyEx661 *[20] and *trIs30 *[28] (Figure 5G-I). We find that both dorsal and lateral body wall muscle morphology is qualitatively normal in *mbl-1 *mutants. Muscles in both wild type and mutant are of similar size and shape.

In *C. elegans*, specialized extensions called muscle arms extend from the muscle to reach motor axons to form NMJs. Therefore, similar to dendritic spines, muscle arms represent postsynaptic components for NMJ formation in *C. elegans*. We found that muscle arms from both the dorsal and lateral muscles in *mbl-1(tm1563) *appear qualitatively normal in size and shape (Figure 5G-J). We further quantified the number of muscle arms from the lateral muscles and found that the number of muscle arms per muscle for wild-type and *mbl-1(tm1563) *animals are not significantly different from each other (3.7 ± 0.5 for wild type and 3.9 ± 0.7 for *mbl-1(tm1563)*) (Figure 5I, J). Taken together, these results demonstrate that the *mbl-1 *mutant phenotype does not affect muscle arm outgrowth or postsynaptic receptor localization, and we hypothesized that the phenotype reflects a defect in synapse formation on the presynaptic side.

### *mbl-1 *functions cell autonomously in DA9

**Figure 6:**
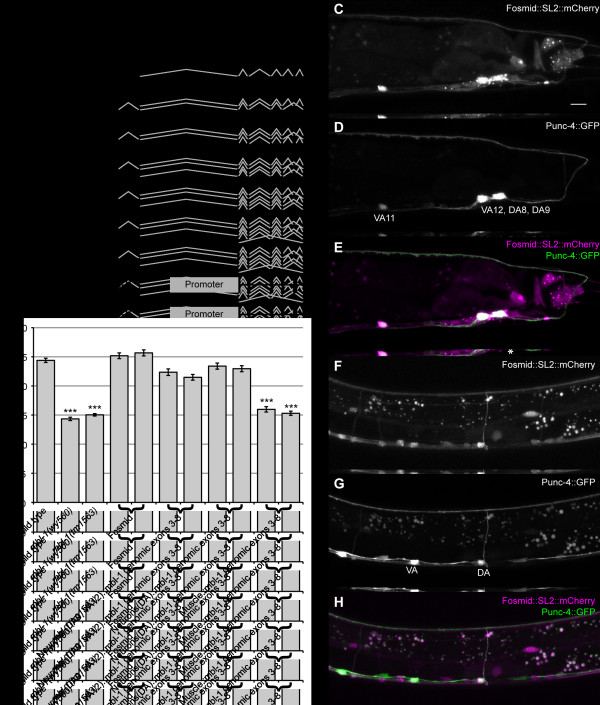
**MBL-1 is required cell autonomously in DA9 and is expressed in many dorsal cord neurons. (A) **Genomic region of the X chromosome including *mbl-1. mbl-1(tm1563) *deletion is indicated above a schematic of genomic region. All cDNA isoforms were reported by Sasagawa *et al. *[31]. Bottom: schematic of genomic rescuing constructs. Black boxes represent exons and thin black lines represent introns. Thin gray lines indicate splicing patterns. **(B) **Quantification of the number of DA9 synapses present in the dorsal cord. Two independent transgenic lines were scored for each rescuing construct. Error bars represent standard error of the mean. ***P < 0.0001, *t*-test. **(C-H) **Endogenous expression of MBL-1 (magenta) and co-localization with A-type motorneurons (green). Transgene used is *wyEx4015*. MBL-1 is expressed in the posterior ventral cord neurons DA8, DA9, VA12, and VA11 (C-E). (F-H) MBL-1 is expressed in A-type motorneurons. DAs can be identified by their commissure crossing the midline of the animals. Scale bar represents 10 μm.

To confirm that mutations in *mbl-1 *cause the synaptic defects observed, we rescued the *mbl-1 *phenotype by injecting a fosmid (WRM0616bE04) that contained genomic DNA encompassing all of the *mbl-1 *exons and introns as well as upstream and downstream non-coding sequences. We found the presence of the genomic fragment completely restored the wild-type number of synapses in a *mbl-1(tm1563) *mutant background (25.2 ± 3.5 and 25.7 ± 3.6; Figure 6B). Rescue was determined by counting the number of visible DA9 synaptic puncta on an epifluorescence microscope.

To determine if the synaptic defects we observe in *mbl-1 *were due to changes in the presynaptic neuron DA9 or to changes in the postsynaptic body wall muscle, we performed cell autonomous rescue experiments (Figure 6). We expressed a genomic fragment including most of the *mbl-1 *ORF under the control of promoters that express in neurons including DA9 (*Pmig-13 *or *Punc-4c*) or body wall muscles (*Phlh-1*) and scored rescue of the DA9 synaptic phenotype in *mbl-1(tm1563) *mutant animals (Figure 6B). While expression of *mbl-1 *in muscle failed to rescue the DA9 synaptic defect (*wyEx4028*, 16.0 ± 3.3; *wyEx4046*, 15.3 ± 2.8), expression of *mbl-1 *in A-type neurons showed significant rescue of the DA9 synaptic defect. *Punc-4c*, a truncated version of the *unc-4 *promoter (unpublished reagent via MV and KS), drives expression in only the DA-type motorneurons. Expression of the *mbl-1 *genomic fragment under the control of the *unc-4c *promoter rescues the DA9 synaptic defect (*wyEx4025*, 23.4 ± 3.8; *wyEx4026*, 23.0 ± 3.7). The *mig-13 *promoter is expressed in DA9, VA12 and other anterior DA neurons and is expressed beginning at embryonic stages. When we used *Pmig-13 *to drive expression of the *mbl-1 *genomic fragment, we found that it also significantly restores the correct number of synapses to the dorsal axon in DA9 (*wyEx4030*, 22.4 ± 4.0; *wyEx4234*, 21.5 ± 3.5). Thus, we conclude that *mbl-1 *is required cell autonomously in the presynaptic neuron to regulate the number of synapses.

Expression of genomic *mbl-1 *in DA9 can also rescue the appearance of ectopic dendritic puncta in *mbl-1(tm1563)*. Expression of the *mbl-1 *genomic fragment under the control of the Pmig-13 or Punc-4c promoters can rescue or partially rescue the appearance of ectopic dendritic puncta. In *mbl-1(tm1563)*, 26% of animals have some dendritic puncta versus 0% and 13% with the Pmig-13 promoter, 4% and 10% with the Punc-4c promoter, versus 18% and 19% with the Phlh-1 promoter.

### *mbl-1 *is expressed in the *C. elegans *nervous system

In order to examine the expression pattern of *mbl-1*, we inserted an SL2::mCherry sequence into the fosmid (WRM0616bE04) containing the *mbl-1 *ORF by homologous recombination. The SL2 sequence acts as a trans-splice acceptor [29] whereby the transcript is expressed under the control of the *mbl-1 *promoter and is later spliced into two separate mRNAs that are translated independently. The recombineered fosmid results in expression of endogenous, untagged MBL-1 and cytoplasmic mCherry, both under the control of the endogenous *mbl-1 *promoter. Examination of transgenic worms carrying the recombineered fosmid reveals that the *mbl-1 *promoter drives expression of mCherry in many cell bodies along the ventral nerve cord (Figure 6C, F). Thin neuronal processes emanate from these cell bodies and fasciculate in both the dorsal and ventral nerve cords.

To determine the neuronal subtypes that express MBL-1, we co-injected the recombineered fosmid with *Punc-4*::GFP, a construct that drives expression of GFP in the A-type (DA and VA) motorneurons [30]. By examining expression patterns of GFP and mCherry, we find that *MBL-1 *is expressed strongly in all of the A-type motorneurons, and also in other neurons in the ventral cord and several unidentified cells in the tail. mCherry expression was seen in embryonic and L1 animals, indicating that *mbl-1 *is expressed early in development. Our expression data are largely consistent with previously reports using a smaller fragment of the *mbl-1 *promoter [31].

**Figure 7:**
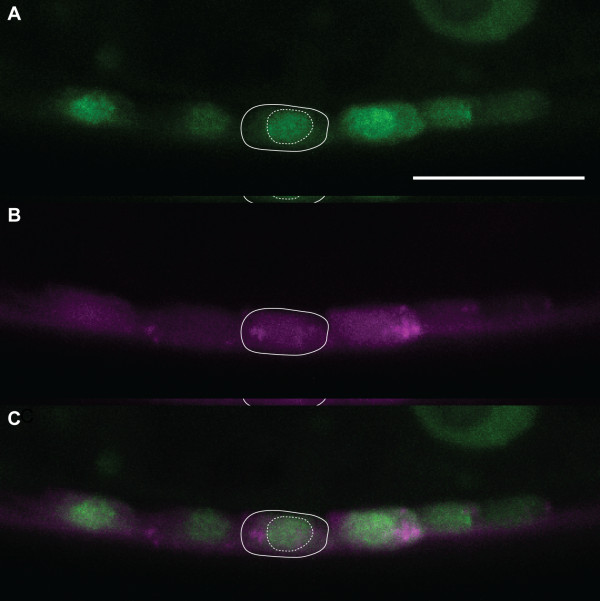
**MBL-1::GFP localizes to the neuronal nucleus. (A, B) **A genomic construct expressing MBL-1::GFP (green) (A) and cytoplasmic mCherry (magenta) (B) under the control of the endogenous *mbl-1 *promoter. **(C) **Merged image of (A) and (B). MBL-1::GFP is concentrated in the nucleus (dashed line) of the cell body (solid line). Scale bar represents 10 μm.

To determine the subcellular localization of *mbl-1*, we created a translational fusion construct by adding a GFP to the carboxyl terminus of *mbl-1*. Expression of MBL-1::GFP was driven by the endogenous *mbl-1 *promoter and we observed that MBL-1::GFP is concentrated in the nucleus of ventral cord neurons, consistent with its putative function in pre-mRNA splicing, which occurs in the nucleus (Figure 7).

### DA synapse density is altered in *mbl-1*

**Figure 8:**
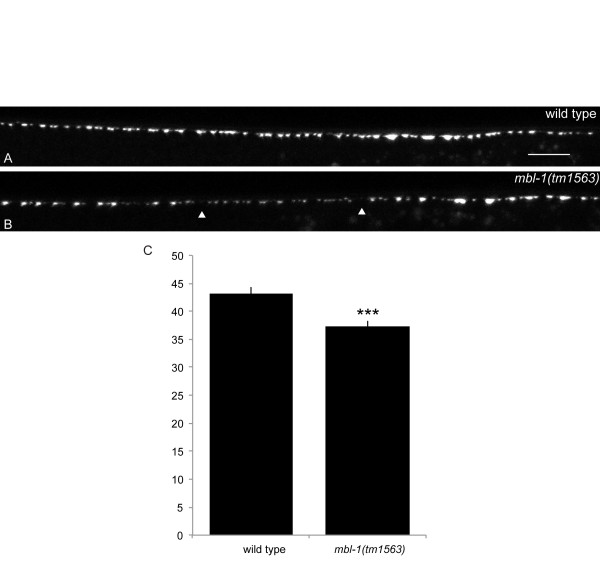
***mbl-1 *reduces DA synapse density. (A, B) **GFP::RAB-3 expressed in all DA neurons (*wyIs222*) in wild type (A) and *mbl-1(tm1563) *(B). Gaps in *mbl-1(tm1563) *are indicated with arrowheads. Image is of the dorsal nerve cord and anterior is to the left. Scale bar represents 10 μm. **(C) **Synapse density difference per 100 μm in wyIs85 and *mbl-1(tm1563)*. Error respresents standard error of the mean. ***P < 0.001, *t*-test.

Because *mbl-1 *is expressed in all A-type motorneurons, we tested whether synapse formation is defective in other neurons of this class. Using a transgenic line that expresses GFP::RAB-3 in all DA neurons under the control of the *unc-4 *promoter, we found that DA synapse density is decreased from 43.2 synapses per 100 μm in wyIs85 to 37.3 synapses per 100 μm in *mbl-1(tm1563) *(*P *< 0.0001) (Figure 8). Individual DA synaptic regions cannot be discerned in this transgenic line, but changes in synapse density arise from significantly large gaps between puncta at regular intervals (Figure 8, arrowheads), rather than a change in the distance between all puncta, implying that all DA synaptic regions are shortened in a similar fashion.

### Loss of *mbl-1 *affects backwards locomotion

**Figure 9:**
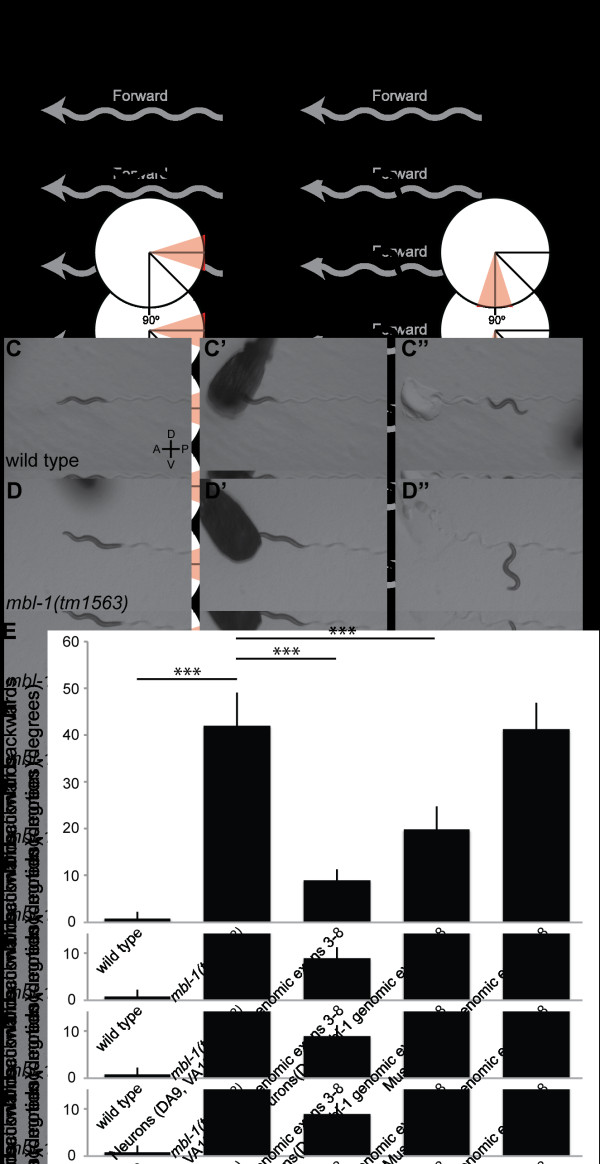
**Locomotion is altered in *mbl-1 mutants*. (A, B) **Cartoon of typical backwards locomotion stimulated by tapping the head for wild type (A) and *mbl-1(tm1563*) (B). The thick gray line and gray arrow indicate tracks and direction of forward movement. The solid black line represents backwards movement. In the diagram, anterior is to the left and dorsal is up. A red triangle on the circular diagram indicates the final angle that would be recorded in this example of the backwards locomotion assay. **(C, D) **Frames from movies of backwards locomotion where the stimulus is delivered at t = 0 s (C', D'). Both (C, D) are at -2 s. (C", D'') Images of the final worm position before re-starting forwards locomotion (C'', t = 2 s; D'', t = 6.3 s). **(E) **Quantification of the angle of backwards locomotion for wild type, *mbl-1(tm1563) *and transgenic strains where *mbl-1 *is expressed under the control of neuronal- or muscle-specific promoters. A positive angle represents a ventral preference during backing. Error bars represent standard error of the mean. ***P < 0.0001, *t*-test.

To understand whether the synaptic phenotypes in the *mbl-1 *mutant affect behavior, we specifically examined the backward locomotion of *mbl-1 *mutants because the A-type motor neurons are involved in backward locomotion [32]. We observed that as *mbl-1 *animals move backwards, they do not back in a straight line as wild-type animals do. Instead, they move backwards more slowly and curve ventrally (Figure 9A-D).

To quantify the backwards locomotion defect, we tapped worms on the head with a platinum wire and recorded the angle of backwards movement where the axis of the preceding forward movement is defined as 0°. A ventral turn during backing is recorded as a positive angle (+) and a dorsal turn as negative (-). The average angle after moving backwards two body lengths for wild type was 0.8 ± 1.5° and 42.0 ± 7.2° for *mbl-1(tm1563) *(Figure 9E). Often the worms will curve 45° per body bend. In addition, *mbl-1(tm1563) *worms often back over many more body lengths than wild-type worms and rarely perform omega bends. This change in backwards locomotion could be explained by the hypocontraction of dorsal body wall muscles in response to a decrease in the density of DA9 motorneuron synapses.

When the same genomic fragment that rescues the DA9 synaptic defect (*mbl-1 *genomic exons 3 to 8) is expressed under the control of the neuronal-specific promoters *Pmig-13 *and *Punc-4c*, we find that the backing behavior is partially rescued. In contrast, when *mbl-1 *is expressed under the control of a muscle-specific promoter (*Phlh-1*), the backing behavior is not rescued. The angles observed after two lengths of backwards movement were 9.0 ± 2.4° for *Pmig-13*, 19.8 ± 5.0° for *Punc-4c*, and 41.3 ± 5.7° for *Phlh-1 *(Figure 9E). These results suggest that the loss of dorsal synapses in DA9 has a measurable affect on backwards locomotion.

## Discussion

In this report we present evidence that the *C. elegans *MBNL splicing regulator *mbl-1 *plays a role in neuromuscular junction synapse formation. A mutation in *mbl-1 *causes a decrease in synaptic density in the cholinergic DA motorneurons and a corresponding defect in the backwards locomotion behavior that these neurons control. We also observed gaps along the dorsal cord where bright synapses would normally be present in wild-type animals. Using a marker that labels only the most posterior DA neuron, DA9, we observed that the ten most distal synapses in DA9 do not form in *mbl-1 *mutant animals. In addition, GFP::RAB-3 localizes ectopically in the dendrite or asynaptic regions of the neuron.

Our data suggest that the defects observed in DA9 are due to the loss of *mbl-1 *specifically in DA9. We observed that *mbl-1 *expression in the presynaptic neuron DA9 was sufficient to rescue both the DA9 synaptic defect and a backwards locomotion defect mediated by DA9. Although muscle defects are seen in mice or *Drosophila *when MBNL1 or Muscleblind is absent [8,10], we observe no change in the muscle acetylcholine receptor ACR-16 localization, nor does expression of an *mbl-1 *transgene in muscles rescue the DA9 synaptic defect. Taken together, these results strongly suggest that *mbl-1 *functions cell autonomously in DA9 to control aspects of synapse formation and behavior.

The mechanism by which *mbl-1 *might be affecting synapse formation is still unknown. Although *C. elegans mbl-1 *itself has not been shown to be a regulator of alternative splicing, it shares a significant amount of homology with human, mouse, and *Drosophila *MBNL sequences, binds to similar repetitive RNA sequences as human MBNL1, and co-localizes with CUG repeat-containing nuclear foci in a *C. elegans *model of DM1 [31,33]. If *C. elegans mbl-1 *is a regulator of alternative splicing, it is likely that the defects we observe in *mbl-1 *mutants are due to changes in the splicing patterns of downstream target genes. Previous studies identified a number of mis-splicing events in MBNL loss-of-function models. Two of these genes, tau/MAPT and the N-methyl-D-aspartate (NMDA) receptor are expressed in the nervous system [34]. We tested if loss of the *C. elegans *genes *ptl-1*/tau or *nmr-1*/NMDA receptor resulted in changes in *C. elegans *NMJ synapse formation. We found that there are no defects in synapse formation in these mutants and the results suggest that the defects we observe in *mbl-1 *are not due to changes in expression levels of these genes.

There are several possible cell functions that are affected downstream of *mbl-1 *and lead to the observed DA phenotype. The loss of the most distal synapses in the DA9 synaptic region and the resulting gaps visible along the dorsal cord could be a result of an alteration in intracellular trafficking dynamics such that the most distal synapses never received a sufficient supply of synaptic vesicle precursor vesicles to build a synapse.

Indeed, alterations in trafficking synaptic vesicle precursors can have dramatic effects on synapse formation. Loss of the *Drosophila *Kinesin-3 *imac *leaves motorneuron axon guidance and morphology unchanged, but causes a dramatic reduction of active zone protein accumulation at presynaptic sites [35]. Mutations in the cargo-binding plexstrin-homology (PH) domain of the *C. elegans *KIF1A homolog *unc-104 *affects both transport of synaptic vesicles to the synapse and motor velocity [36,37]. The defect we observe in *mbl-1 *could be caused by a small change in motor activity, which reduces cargo-binding capacity or decreases motor velocity, and thus the efficiency of transport.

We know of several extrinsic cues that are required to specify the DA synaptic regions. The posterior border of the DA9 synaptic region is established via a gradient of the secreted Wnt protein *lin-44 *and its receptor Frizzled (*lin-17*), expressed in DA9 [20]. Our lab has also identified binding between plexins and semaphorins as essential to establishing the synaptic domains for each of the DA neurons, a process referred to as 'tiling' (KM and KS, unpublished data). Mutations in the plexin *plx-1 *and the semaphorin *smp-1 *lead to overlap between individual DA synaptic regions, though the mechanism by which they function is unknown. In *mbl-1 *we observe gaps, rather than overlaps, between individual DA neurons, but it is possible that *mbl-1 *regulates some aspect of the tiling process and effectively shortens synaptic domains.

## Materials and methods

### Strains and genetics

The wild-type reference was the N2 Bristol strain. Strains were maintained by standard methods [38]. *mbl-1(wy560) *was originally obtained from the Deletion Consortium as strain RB771. The *hum-4(ok550) *deletion mutation was outcrossed away from the *mbl-1(wy560) *allele and the resulting strain is TV7337. *mbl-1(tm1563) *was obtained from the Mitani lab.

### Cloning and constructs

The following plasmids and transgenic strains were generated using standard techniques: *wyIs85 (Pitr-1 pB::GFP::rab-3), wyIs92 (Pmig-13::snb-1::YFP), wyIs222 (Punc-4::GFP::rab-3), wyEx661 (pRF4(rol-6(su1006)), Pmyo-3::myr::mCherry, Pmig-13::SNB-1::YFP), wyEx802 (pRF4(rol-6(su1006)), Pmyo-3::acr-16::GFP, Pmig-13::mCherry::rab-3), wyEx1902(Pitr-1pB::mCherry), wyEx2055 (Pitr-1::GFP::syd-2, Pitr-1::mCherry::rab-3), wyEx3709 (Pmig-13::unc-10::GFP, Pmig-13::mCherry::rab-3), wyEx4015 (WRM0616bE04::SL2::mCherry, Punc-4::GFP), wyEx4029 (WRM0616bE04 *injected at 5 ng/μl), *trIs30 (Phim-4p::MB::YFP, Phmr-1b::DsRed2, Punc-129nsp::DsRed2)*. Rescue of *mbl-1(wy560) *by *Pmig-13::mbl-1 genomic exons 3-8 *involved injection of plasmid KAS138 into the mutant animals at 10 ng/μl. The co-injection markers *Podr-1::GFP *and *Podr-1::dsRED *(20 ng/μl) were used for most strains. *wyEx661 *and *wyEx802 *used pRF4(*rol-6(su1006)*) at 50 ng/μl.

Rescuing constructs were constructed by using the primers 5'-CAAGGGGGCGCGCCATGTTCGACGAAAACAGTAATGCCG-3' and 5'-GGTACCCTAGAATGGTGGTGGCTGCATGTA-3' to amplify a 6.6-kb fragment called '*mbl-1 *genomic exons 3-8'. This fragment was inserted into a plasmid carrying the appropriate promoter using AscI and KpnI restriction sites. Strains constructed were the following: *wyEx4030 and wyEx4234 (Pmig-13::mbl-1 genomic exons 3-8), wyEx4025 and wyEx4026 (Punc-4c::mbl-1 genomic exons 3-8), wyEx4028 and wyEx4046 (Phlh-1::mbl-1 genomic exons 3-8)*.

### Imaging

Epifluorescence imaging was conducted on a Zeiss AxioSkop or AxioVivison epifluorescent microscope with a mercury or X-cite light source. To create composite images of DA9 synaptic regions, ten confocal images were obtained for each genotype using the same imaging parameters on a Zeiss LSM 710 confocal microscope. Images were processed in ImageJ [39] using the Straighten.jar plugin [40]. A line was drawn from the point at which DA9 reaches the dorsal cord to the most anterior part of DA9 captured in the image. After isolating all of these images, we aligned them along their anteroposterior axes and assembled them into a composite image in Adobe Illustrator. Fluorescence intensities were graphed using values generated with the ImageJ function 'Plot profile'.

### Backwards locomotion assay

To assay backwards locomotion, worms were transferred onto freshly spotted plates 20 to 60 minutes before the assay. Individual worms that had just completed forward locomotion of at least two body lengths were tapped and the resulting backing angle was observed and recorded. The assay was repeated three times for each individual and an average of the three trials was taken. Images of the assay were captured with a Q-Imaging Retiga 2000R camera mounted on a Zeiss Stemi SV11 dissection microscope.

### Identification of the wy560 deletion

We performed snip-SNP mapping using SNPs from the Hawaiian strain [23] to identify a 1.5-Mb region on the extreme right end of the X chromosome containing the mutant allele. Deep sequencing on a Solexa Illumina sequencer revealed that the *wy560 *deletion spans from 16,983,396 to 17,048,700 on the X chromosome. This 65-kb deletion eliminates eight genes. Only the fosmid WRM0616bE04 rescued the DA9 synaptic phenotype.

## Abbreviations

bp: base pair; DM1: myotonic dystrophy type 1; DNC: dorsal nerve cord; GFP: green fluorescent protein; MBNL: Muscleblind-like; NMJ: neuromuscular junction; ORF: open reading frame; SNP: single-nucleotide polymorphism.

## Competing interests

The authors declare that they have no competing interests.

## Authors' contributions

KAS and KS designed the experiments and KAS performed the experiments. KAS and GJW mapped the *mbl-1(wy560) *allele. MST contributed the data for *ptl-1 *and *nmr-1*. KAS and KS wrote the paper. All authors read and approved the final manuscript.
